# Suprascapular Notch Asymmetry: A Study on 311 Patients

**DOI:** 10.1155/2014/196896

**Published:** 2014-05-12

**Authors:** Michał Polguj, Marcin Sibiński, Andrzej Grzegorzewski, Piotr Grzelak, Ludomir Stefańczyk, Mirosław Topol

**Affiliations:** ^1^Department of Angiology, Medical University of Łódź, Ulica Narutowicza 60, 90-136 Łódź, Poland; ^2^Clinic of Orthopedic and Pediatric Orthopedics, Medical University of Łódź, Ulica Drewnowska 75, 91-002 Łódź, Poland; ^3^Department of Radiology, Medical University of Łódź, Ulica Kopcińskiego 22, 90-153 Łódź, Poland; ^4^Department of Normal and Clinical Anatomy, Medical University of Łódź, Ulica Narutowicza 60, 90-136 Łódź, Poland

## Abstract

The most important risk factor of suprascapular nerve entrapment is probably the shape of the suprascapular notch (SSN). The aim of the study was to perform a radiological study of the symmetry of SSN. Included in the study were 311 patients (137 women and 174 men) who underwent standard computed tomography investigation of the chest. A total of 622 computed tomography scans of scapulae were retrospectively analyzed to classify suprascapular notches into five types. Suprascapular notch was recognized as a symmetrical feature in 53.45% of the patients. Symmetry was more frequently seen in females (54.0% versus 52.9%), but not to any significant degree (*P* = 0.8413). Type III was the most commonly noted symmetrical feature (66.9%) and type II was less common (0.6%). Type III was the most symmetrical type of suprascapular notch, occurring significantly more often as a symmetrical feature in comparison with type I (*P* < 0.0001), type II (*P* = 0.00137), or type IV (*P* = 0.001). Our investigation did not show that the suprascapular notch is a symmetrical feature. However, symmetry was recognized more frequently in the case of type III SSN. No significant differences in symmetry were found with regard to sex.

## 1. Introduction

The suprascapular notch (SSN) is a depression on the superior border of the scapula covered by the superior transverse scapular ligament (STSL) [1]. This tunnel forms a passage for the suprascapular nerve (SN). As the SSN is the most common site of both injury and compression of the SN, the region is of paramount importance in the etiopathogenesis of suprascapular neuropathy [2–4]. Due to the higher frequency of this pathology observed in patients aged under 38, it is also important from a demographic point of view [1, 4].

Knowledge of the morphology of the suprascapular notch is extremely important because its shape is the most important risk factor in the etiopathology of suprascapular nerve entrapment formation [5–8]. It is also particularly essential in clinical practise due to various techniques associated with arthroscopic SN decompression [9–11]. The aim of the study was to perform a large-scale, based on specific geometrical parameters of the SSN, radiological study of the symmetry of SSN.

## 2. Materials and Methods

A total of 359 consecutive patients who underwent standard chest computed tomography between December 2007 and February 2011 participated in the study. From the studied group, 48 patients were excluded due to metastases to bone, deformations of the scapulae, or poor image quality. Multidetector computed tomography (MDCT) images of 311 adult patients (137 women and 174 men; mean age 62 years) were retrospectively analysed, with particular focus on the superior border of the scapula, to detect anatomical variations of the suprascapular notch. The research project was approved by the Bioethics Commission of the Medical University (Protocol number RNN/12/10/KE).

A breath-hold dual-phase helical computed topography was performed with a 32-row MDCT scanner (Toshiba Aquilion 32; Toshiba Medical System, Japan; slices thickness 1.0 mm, kVp 120, mAs 225, 500-ms tube rotation). All shoulders were analyzed with postprocessing tools; maximum intensity projection and multiplanar reconstruction images were obtained along the coronal and sagittal planes, and three-dimensional volume rendering reconstruction was obtained for the scapulae.

**Figure 1:**
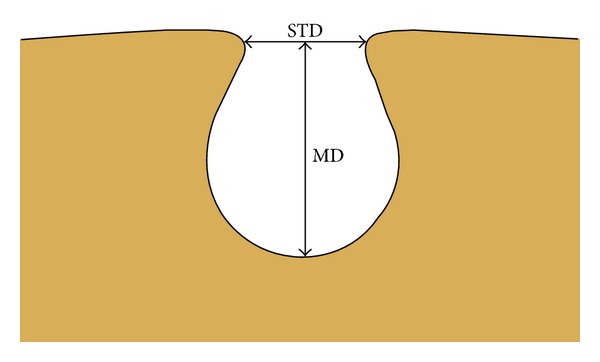
Schematic arrangements of the measurements of suprascapular notch. MD: maximal depth; STD: superior transverse diameter.

The following measurements of the suprascapular notch using Vitrea 2 system software (Vital Images, Plymouth, MN, USA) were collected (Figure 1):

1. the maximal depth (MD): the maximum value of the longitudinal measurements taken in the vertical plane from an imaginary line between the superior corners of the notch to the deepest point of the suprascapular notch;
2. the superior transverse diameter (STD): the maximal value of the horizontal measurements taken in the horizontal plane between the corners of the SSN on the superior border of the scapula.

**Figure 2:**
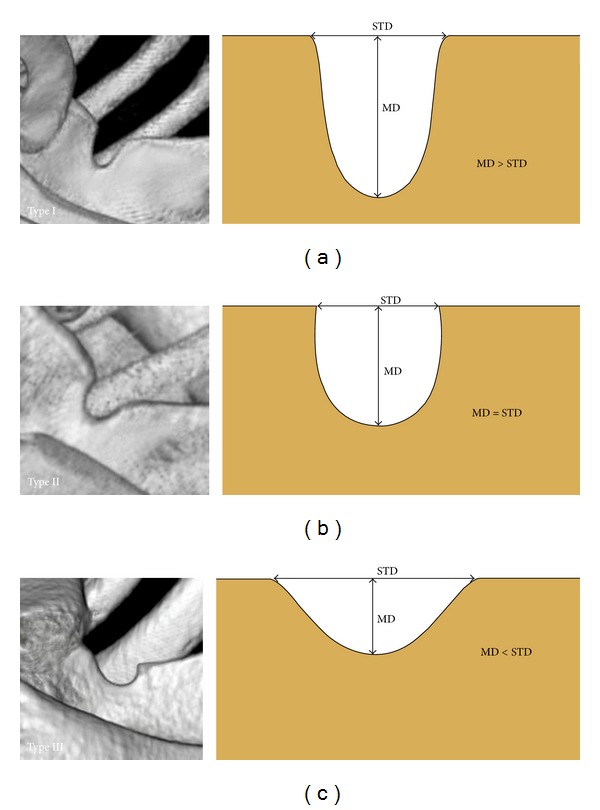
Types I–III of the suprascapular notch (three-dimensional volume rendering MDCT). (a) Type I, (b) type II, and (c) type III. MD: maximal depth; STD: superior transverse diameter.

**Figure 3:**
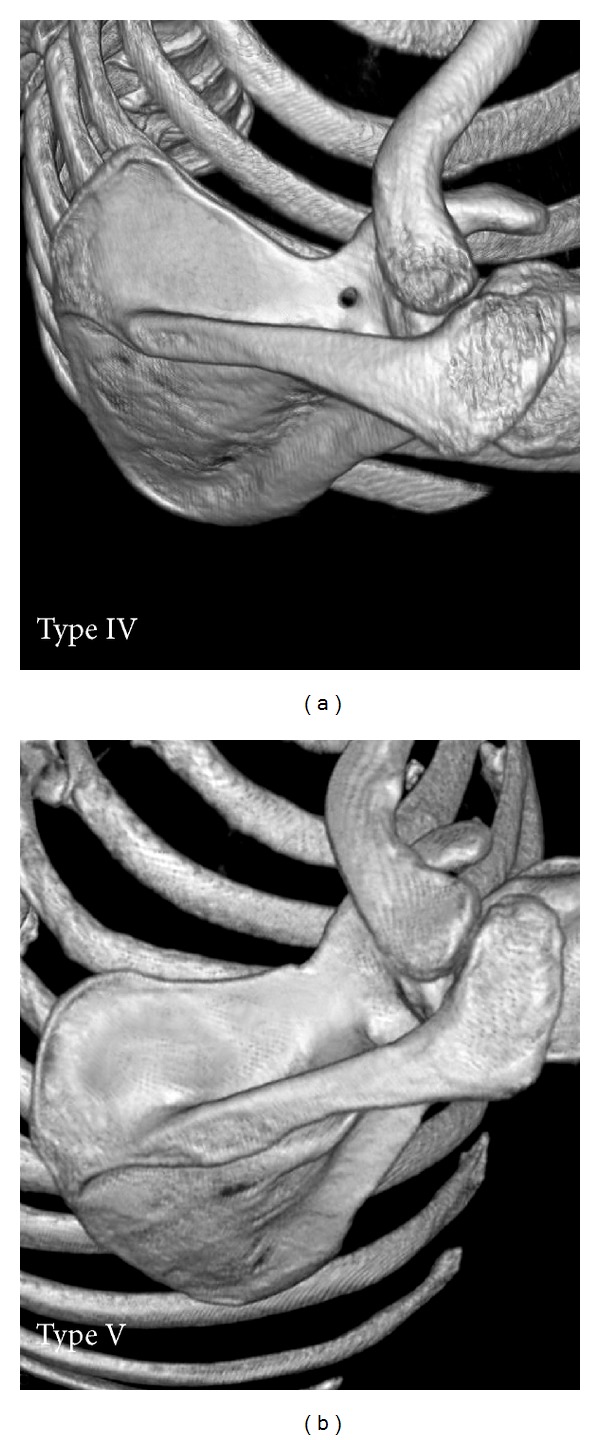
Types IV-V of the suprascapular notch (three-dimensional volume rendering MDCT). (a) Type IV; (b) type V.

The shape of suprascapular notch was determined by using the fivefold, simplified classification system defined by Polguj et al. (2011) [12]. The classification is as follows: MD was longer than STD in type I (Figure 2(a)), MD and STD are equal in type II (Figure 2(b)), STD is longer than MD in type III (Figure 2(c)), a suprascapular foramen with a bony bridge joins the corners of the SSN in type IV (Figure 3(a)), and a discrete notch is presented in type V (Figure 3(b)). Each of the computed tomography scans of the shoulder area was carefully observed simultaneously by two investigators.

Statistical analysis for all parameters was performed with the use of Statistica 10 software (StatSoft Polska, Cracow, Poland). The normality of data distribution was checked by means of the Shapiro-Wilk test. The statistical difference between the symmetry of the type of suprascapular notch in both sexes was examined using the Chi^2^ test. In statistical analysis, *P* < 0.05 was considered significant. The Chi^2^ or Fisher's exact test was used when comparing the symmetry of the suprascapular notch between sexes and different types of suprascapular notch. For multiple testing, the Bonferroni correction was applied.

## 3. Results

The suprascapular notch was recognized as a symmetrical feature in 166 of 311 patients (53.45%): 74 women and 92 men. Although symmetry was more frequently seen in females (74/137, 54.0% versus 92/174, 52.9%), this was not significantly different to the males according to the Chi^2^ test (*P* = 0.8413).

**Figure 4:**
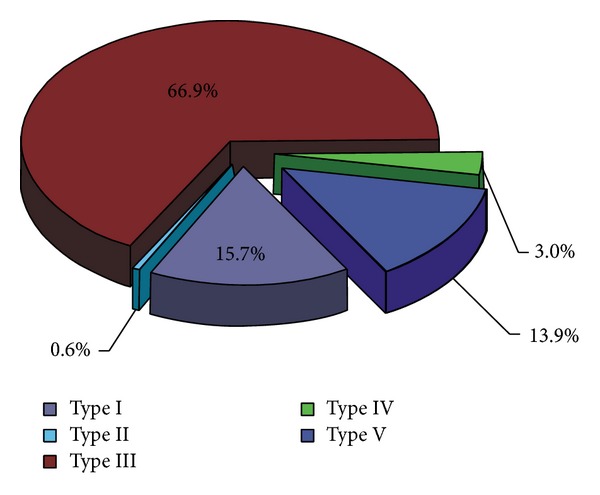
The frequency of the suprascapular notch types as symmetrical feature.

Among the scans of scapulae examined, the most common symmetrical type was type III (111/166, 66.9%) (Figure 2(c)). Type II (1/166, 0.6%) (Figure 2(b)) was the least. The frequency of symmetry of type I was 15.6% (26/166) (Figure 2(a)). Type IV (scapulae with bony foramen) was noted to occur symmetrically in 5 of the 166 patients (3.0%) (Figure 3(a)). The frequency of symmetry of type V (discrete notch) was 13.9% (in 23 from 166 patients) (Figure 3(b)). The distributions of types of SSN as symmetrical features are presented in Figure 4.

**Figure 5:**
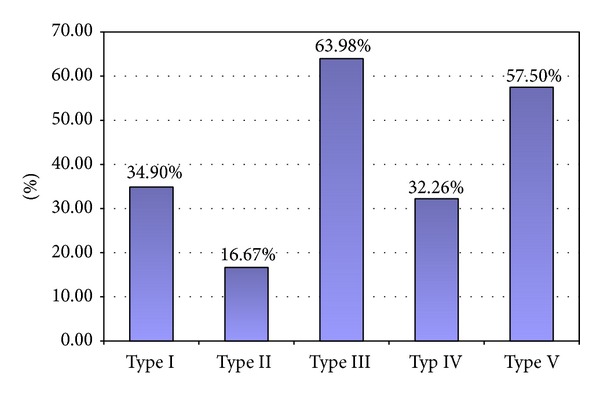
The distributions of symmetry of types of SSN taking into consideration each type independently.

Taking into consideration each type of suprascapular notch independently, type III as a symmetrical feature was recognized in 222 from 347 scapulae (63.98%) with this shape of SSN. Analogically, the frequency of symmetry of other types was 34.9% (52 from 149 scapulae with type I), 16.67% (2 from 12 scapulae with type II), 32.26% (10 from 31 scapulae with type IV), and 57.5% (46 from 80 patient with type V) (Figure 5).

According to the statistical analysis, symmetry was noted significantly more often in type III than in types I (*P* < 0.0000), IV (*P* = 0.001), and II (*P* = 0.00137). Type III occurred more often as a symmetrical feature than type V, but no statistical significance was identified (*P* = 0.28).

## 4. Discussion

The most important finding of this study was the quantitative analysis of the symmetry of the suprascapular notch. According to our study, the suprascapular notch is not a symmetrical feature because only little more than a half of studied patients were found to have the same type bilaterally. However, some shapes of the suprascapular notch (especially, type III) were recognized to be symmetrical more frequently. Such observation is interesting from morphological point of view and may be also clinically helpful in open and endoscopic procedures of suprascapular nerve decompression. This study is also based on imaging techniques and presents usefulness of specific geometrical parameters in classification of the SSN morphological variations. Such a method is simple, reproducible, and clearly distinguishes each type. As suprascapular nerve entrapment is a rare condition, such parameters are especially clinically important because they confirm their practical viability in multicenter investigations focused on this pathology.

According to Dunkelgrun et al. [5] and Natsis et al. [6], the shape and size of the suprascapular notch are the most important factors in the etiopathology of suprascapular nerve entrapment formation. Rengachary et al. [7] note that a narrow SSN may predispose a patient to suprascapular neuropathy formation. Also, Antoniadis et al. [8] state that a V-shaped notch is more likely to be connected with this pathology. The influence of the shape of SSN is also supported by the* sling effect*, the first theory explaining suprascapular nerve entrapment proposed in 1979 by Rangrery et al. [7]. It posits that, during motions of the upper limb, the suprascapular nerve can be pressed againstthe sharp bony margin of the SSN. Repeated kinking irritates the nerve and may induce microtrauma that result in neuropathy [7]. When the SN travels through a “deep and narrow” SSN, like in our study type I, it may be more predisposed to injury by the sharp bony walls.

Knowledge of the suprascapular notch symmetry is also important because suprascapular nerve entrapment syndrome may be found bilaterally [13, 14]. Aydin et al. [13] present the case of a twenty-year-old man with a history of bilateral shoulder pain associated with weakness and atrophy of the supraspinatus and infraspinatus muscles. Also, Alon et al. [14] describe the rare case of a young woman who suffered from suprascapular nerve entrapment of the right side and two years later developed the same syndrome on the left. Bilateral anomalous bifid superior transverse scapular ligaments were found.

Several other morphological variations of structures in the suprascapular region should be taken into consideration as important risk factors in the formation of suprascapular nerve entrapment, such as a multiband superior transverse scapular ligament [15], hypertrophied subscapular muscle [16], a double suprascapular foramen [17], the spinoglenoidal ligament [18], a completely ossified superior transverse scapular ligament [19], or topography of the suprascapular artery [20, 21].

The newest quantitative classification system was used for this study [12] for a number of reasons. Not only is it simple and reproducible, but also it is based on both the shape of the superior border of the scapula and two main geometrical measurements of the suprascapular notch. Currently the gold standard for the generation of 3D models is computed tomography [22]. Three-dimensional volume rendering of bone models offers a satisfactory source of information for morphological studies and investigations of biomechanical characteristics of bones in complex anatomic regions [23].

According to Paraskevas et al. (2008) [24] the shape of the acromion was symmetrical in 65.9% of cases, and their results reveal no correlation between shape and gender. However, Shi et al. [25] confirm that there is excellent side-to-side symmetry in glenoid shape and demonstrate the value of the use of a contralateral glenoid as a marker of initial glenoid size in patients with unilateral glenoid bone loss or deformation.

The nonspecific symptoms of suprascapular nerve entrapment can result in late diagnosis when atrophy of the supraspinatus and infraspinatus muscles is recognized. In accordance with Gosk et al. [3], the outcome of surgery depends on the length of time between symptom onset and the surgery itself and the pathology underlying the nerve compression. All studies on anatomical variations of structures located at the suprascapular region should prove their value in the diagnosis and treatment of this pathology. According to a current bibliography search, only a few studies based on imaging techniques have been performed on anatomical structures in the suprascapular region [26–28]. Our study has potential clinical benefits because it may prove essential for surgeons performing SN decompression, especially by means of endoscopic techniques [9–11].

The limitation of this study is that data was determined only retrospectively. Because of the radiation dose involved, the acquisition of data sets from patients is not ethically justifiable. Therefore we had no information describing symptoms of suprascapular nerve entrapment. Also movement artifacts may make standardized measurement difficult. Moreover, in our analysis, all CT investigations with poor image quality were excluded from the study.

## 5. Conclusion

Our investigation does not demonstrate that the suprascapular notch is a symmetrical feature because only little more than half of studied patients were found to have the same type bilaterally. However, some shapes of the suprascapular notch were recognized to be symmetrical more frequently, particularly type III. No significant differences in symmetry were found with regard to sex.
